# DNA methylation silencing of microRNA gene methylator in the precancerous background mucosa with and without gastric cancer: Analysis of the effects of *H*. *pylori* eradication and long-term aspirin use

**DOI:** 10.1038/s41598-019-49069-1

**Published:** 2019-08-29

**Authors:** Jiro Watari, Chiyomi Ito, Tadakazu Shimoda, Toshihiko Tomita, Tadayuki Oshima, Hirokazu Fukui, Kiron M. Das, Hiroto Miwa

**Affiliations:** 1 Division of Gastroenterology, Department of Internal Medicine, Hyogo College of Medicine, Nishinomiya, Japan; 2 Division of Pathology, Shizuoka Cancer Center, Shizuoka, 411-8777 Japan; 3 Division of Gastroenterology and Hepatology, Departments of Medicine and Pathology, Robert Wood Johnson Medical School, Rutgers, Cancer Institute of New Jersey, New Brunswick, New Jersey 08903 United States

**Keywords:** Predictive markers, Gastric cancer, Molecular medicine, Cancer epigenetics, Risk factors

## Abstract

The risk of gastric cancer (GC) declines after *Helicobacter pylori* (*H*. *pylori*) eradication and long-term aspirin use. We evaluated the effects of *H*. *pylori* eradication (Cohort 1) and aspirin use (Cohort 2) on the methylation of microRNAs (miRNAs), such as *miR-*3*4c*, *miR-124a-3*, *miR-*129*-*2, and *miR-1*3*7*, in the gastric mucosa with and without GC, i.e., in atrophic mucosal glands without intestinal metaplasia (non-IM) and intestinal metaplastic glands (IM). DNA was isolated from non-IM and IM separately using laser caption microdissection. In Cohort 1, *H*. *pylori* eradication was associated with a significant reduction of *miR-124a-3* methylation only in non-IM, but not in IM. *miR-129-2* methylation in non-IM may be a surrogate marker of GC in *H*. *pylori*-infected patients. In Cohort 2, aspirin did not reverse miRNA methylation in either non-IM or IM, irrespective of *H*. *pylori* infection. *miR-129-2* methylation in non-IM was an independent predictive marker of GC in *H*. *pylori*-infected but not -eradicated patients. These results indicate that *H*. *pylori* eradication and aspirin use were less effective for improving methylation in IM than in non-IM; thus, these interventions are recommended at an early stage prior to the development of IM to prevent GC development. In addition, the effects of the interventions were not uniform for each miRNA gene.

## Introduction

Gastric cancer (GC) is a leading cause of cancer death worldwide^1^, with *Helicobacter pylori* (*H*. *pylori*) considered to be a main risk factor^2^. In Correa’s hypothesis, chronic infection with *H*. *pylori* has been postulated to develop over decades into chronic gastritis, gastric atrophy, usually intestinal metaplasia (IM), dysplasia, and GC^2^.

To date, some meta-analyses have shown that *H*. *pylori* eradication reduced the risk of GC not only in patients with chronic gastritis but also in patients who underwent endoscopic resection for early GC^3–8^. On the other hand, although several studies have been done regarding the effect of *H*. *pylori* eradication in preventing metachronous GC, the results remain controversial: some studies have shown that *H*. *pylori* treatment led to a lower incidence of metachronous GC^9–11^ and others have not^12–14^. Long-term studies from Japan showed that even after *H*. *pylori* eradication, the risk of developing GC remains, and the risk increases under the preneoplastic conditions of the background mucosa, i.e., gastric atrophic mucosa and IM^15,16^. These results indicate that *H*. *pylori* eradication treatment may reduce the risk of GC, though it may not abolish the risk.

Aspirin also has protective effects against certain cancers. Recent reports including meta-analyses have shown that long-term aspirin use (for at least more than 3 years) was associated with a reduced GC risk^17–20^. However, the majority of these studies included both *H*. *pylori*-infected and *H*. *pylori*-negative subjects. Cheung *et al*. showed that the risk significantly decreased with increasing frequency, duration, and dose of aspirin after *H*. *pylori* eradication^21^.

GC develops through the accumulation of genetic and epigenetic alterations. Many studies have reported that several epigenetic alterations, including promoter hypermethylation of multiple tumor-related genes, are associated with GC and precancerous conditions of the stomach that occur in the context of *H*. *pylori* infection^22–34^. These reports demonstrated that *H*. *pylori* eradication led to a decrease in methylation levels in some genes^22–27^, suggesting that the reduction of gene methylation reversed *H*. *pylori*-induced gastric carcinogenesis. However, there are no studies except ours^33,34^ and a study by Huang *et al*.^32^ in which molecular events were analyzed in atrophic mucosal glands without IM (non-IM) and with IM separately using laser capture microdissection (LCM); thus, the actual effects of eradication therapy on molecular alteration in patients with the precancerous conditions are not well known.

Currently, microRNAs (miRNAs) are considered to be actively involved in the development, differentiation, and pathogenesis of various malignancies. By comparing the miRNA expression profiles in tumor tissues versus adjacent non-tumor tissues, distinct patterns of up- or down-regulation of miRNAs were found in different types of cancers^35^. Therefore, these cancer-specific miRNA expressions could be used as molecular biomarkers for GC. Indeed, several studies showed that the dysgeneration of some miRNAs by methylation highlights the useful biomarkers of GC development^29–31,35–41^. Japanese investigators have recently reported that *miR-124a-3* and *miR-34b/c* methylation are informative markers for predicting the risk of metachronous GC in patients after the endoscopic resection of early GC^29,36,37^. However, we reported that the incidences of the methylation of *miR-124a-3* and *miR-34c* were mostly observed in IM, with very few in non-IM^33,34^. Thus, our previous results indicate that the methylation of these miRNA genes might be a specific marker expressed in IM and might not necessarily be a risk marker for GC.

Our aims in this study were: 1) to investigate the methylation changes of several miRNAs related to gastric carcinogenesis in patients before and after *H*. *pylori* eradication in patients not taking low-dose aspirin (LDA) or nonsteroidal anti-inflammatory drugs (NSAIDs) (Cohort 1); and 2) to examine the effects of LDA/NSAIDs on the methylation status of those miRNAs before and after *H*. *pylori* eradication in patients who had regularly taken LDA/NSAIDs on a long-term basis (≥3 yr) (Cohort 2) while exhibiting a precancerous condition, i.e., non-IM or IM.

## Results

### Patients’ characteristics

The characteristics of the patients are shown in Table 1. In both Cohorts 1 and 2, there were no significant differences in median age or sex between the atrophic gastritis (AG) and GC groups in *H*. *pylori*-infected and -eradicated patients. However, the number of IM samples was significantly higher in the *Hp*−/GC and *Hp*−/LDA/GC groups than in the *Hp*+/AG and *Hp*+/LDA/AG groups (*p* = 0.01 and *p* = 0.0004, respectively).

**Table 1:** Patients’ characteristics.

Cohort 1	*Hp*+/AG	*Hp*+/GC	*p*–value	*Hp−*/AG	*Hp*−/GC	*p*–value	*Hp*+/AG vs *Hp*−/AG	*Hp*+/GC vs *Hp*−/GC
	(n = 21)	(n = 26)		(n = 30)	(n = 27)		*p*–value	*p*–value
Past eradication (y) (1^st^–3^rd^ quartile)	—	—	—	5 (3–7)	5 (4–9)	0.65	—	—
Median age (y) (1^st^–3^rd^ quartile)	65.0 (59.5–73.0)	69.0 (65.0–72.3)	0.22	66.5 (54.5–75.3)	68.0 (64.0–73.0)	0.24	0.72	0.68
Male:Female	13:8	18:8	0.60	14:16	19:8	0.07	0.28	0.93
Total no. of samples	62^a^	75^b^		90	80^c^			
Non-IM samples	41	40	0.13	67	45	0.01	0.27	0.72
IM samples	21	35		23	35			
**Cohort 2**	***Hp*** **+/LDA/AG**	***Hp*** **+/LDA/GC**		***Hp*** **−/LDA/AG**	***Hp*** **−/LDA/GC**		***Hp*** **+/LDA/AG** **vs** ***Hp*** **−/LDA/AG**	***Hp*** **+/LDA/GC** **vs** ***Hp*** **−/LDA/GC**
	(**n** = **3**)	(**n** = **11**)	***p*** **–value**	(**n** = **21**)	(**n** = **11**)	***p*** **–value**	***p*** **–value**	***p*** **–value**
Median period of prior aspirin use (y) (1^st^–3^rd^ quartile)	10 (6–11)	6 (3–7)	0.19	6 (4–7)	6 (4–7)	0.54	0.07	0.90
Median age (y) (1^st^–3^rd^ quartile)	75.0 (74–76)	73.0 (67.5–80)	0.19	77.0 (75–78)	81.0 (71–82)	0.18	0.72	0.68
Male:Female	3:0	10:1	1	12:9	10:1	0.11	0.28	0.93
Total no. of samples	9	33		63	32 ^d^			
Non-IM samples	3	13	1	53	16	0.0004	0.27	0.72
IM samples	6	20		10	16			

^a^One sample, ^b^three samples, ^c^one sample, and ^d^one sample could not be analyzed because they were too small.

*Hp*, *H*. *pylori*; AG, atrophic gastritis; GC, gastric cancer; IM, intestinal metaplasia; LDA, low–dose aspirin.

#### Cohort 1

Molecular events in non-IM: The *miR-124a-3* and *miR-137* methylation rates were significantly lower in the *Hp*−/AG group than in the *Hp*+/AG group (*p* < 0.0001 and *p* = 0.06, respectively) (Table 2), thus indicating the effects of *H*. *pylori* eradication. Multivariate analysis showed that *H*. *pylori* eradication was associated with a significant reduction of *miR-124a-3* methylation [odds ratio (OR): 0.03, 95% confidence interval (CI): 0.004–0.27, *p* = 0.002]. Similarly, the incidences of *miR*-*34c*, *miR*-*124a*-*3*, and *miR*-*129*-*2* methylation were significantly lower in the *Hp*−/GC group than in the *Hp*+/GC group (*p* = 0.005, *p* = 0.0005, and *p* = 0.01, respectively), and *H*. *pylori* eradication was significantly associated with a reduction of only *miR*-*124a*-*3* methylation (OR: 0.16, 95% CI: 0.04–0.65, *p* = 0.01) in a multivariate analysis.

**Table 2:** Comparison of molecular alterations in non-IM between patients with and without GC in *H*. *pylori*-infected and -eradicated patients (Cohort 1).

	*Hp*+/AG group		*Hp*−/AG group		*p*–value	Multivariate analysis		
	(%)	(*n*)	(%)	(*n*)		OR	95% CI	*p*–value
*miR–34c*	0	(0/41)	0	(0/67)	1	—	—	—
*miR–124a–3*	31.7	(13/41)	1.5	(1/67)	<0.0001	0.03	0.004–0.27	0.002
*miR–129–2*	10.0	(4/40)	7.5	(5/67)	0.73	—	—	—
*miR–137*	10.0	(4/40)	1.5	(1/67)	0.06	0.17	0.016–2.03	0.17
	***Hp*** **+/GC group**		***Hp*** **−/GC group**		***p*** **–value**	**Multivariate analysis**		
*miR–34c*	27.5	(11/40)	4.4	(2/45)	0.005	0.20	0.04–1.07	0.61
*miR–124a–3*	40.0	(16/40)	6.7	(3/45)	0.0005	0.16	0.04–0.65	0.01
*miR–129–2*	40.0	(16/40)	15.6	(7/45)	0.01	0.34	0.11–1.05	0.06
*miR–137*	12.5	(5/40)	4.4	(2/45)	0.25	—	—	—
	***Hp*** **+/AG group**		***Hp*** **+/GC group**		***p*** **–value**	**Multivariate analysis**		
*miR–34c*	0	(0/41)	27.5	(11/40)	0.0002	1593533.79	1.871E–290	1.357E302
*miR–124a–3*	31.7	(13/41)	40.0	(16/40)	0.49	—	—	—
*miR–129–2*	10.0	(4/40)	40.0	(16/40)	0.004	5.21	1.46–18.60	0.01
*miR–137*	10.0	(4/40)	12.5	(5/40)	1	—	—	—

IM, intestinal metaplasia; *Hp*, *H*. *pylori*; AG, atrophic gastritis; GC, gastric cancer; OR, odds ratio; CI, confidence interval.

**Figure 1:**
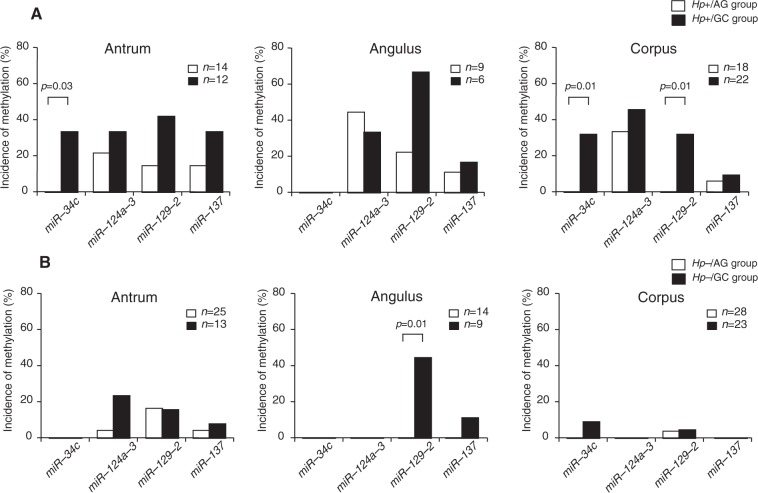
Incidences of miRNA methylation in non-IM in three different parts of the stomach in patients not taking LDA/NSAIDs. (**A**) In *H*. *pylori*-infected patients, the *miR-34-c* methylation rate in the antrum (*p* = 0.03) and corpus (*p* = 0.01) was significantly higher in the *Hp*+/GC group than it was in the *Hp*+/AG group. Also, *miR-129-2* methylation in non-IM in the corpus was more frequently identified in the *Hp*+/GC group than in the *Hp*+/AG group (*p* = 0.01). (**B**) In *H*. *pylori*-eradicated patients, the *miR-129-2* methylation rate in the angulus in the *Hp*−/GC group was significantly higher than that in the *Hp*−/AG group (*p* = 0.01). LDA, low-dose aspirin; NSAID, nonsteroidal anti-inflammatory drug.

In *H*. *pylori*-infected AG patients, the incidence of *miR-124a-3* methylation in the *Hp*+/AG group was 31.7% and that of the methylation of other miRNA genes was very low (Table 2). However, the *miR-34c* and *miR-129-2* methylation rates were significantly higher in the *Hp*+/GC group than in the *Hp*+/AG group (*p* = 0.0002 and *p* = 0.004, respectively). Multivariate analysis showed that *miR-129-2* methylation in non-IM was significantly associated with GC (OR: 5.21, 95% CI: 1.46–18.60, *p* = 0.01). When comparing the methylation rates of these miRNAs in non-IM among the three different parts of the stomach, the *miR-34-c* methylation rate in the *Hp*+/GC group was significantly higher in the antrum (*p* = 0.03) and corpus (*p* = 0.01) than in the *Hp*+/AG group (Fig. 1A). Also, *miR-129-2* methylation in non-IM in the corpus was more frequently identified in the *Hp*+/GC group than in the *Hp*+/AG group (*p* = 0.01) (Fig. 1A).

In *H*. *pylori*-eradicated cases, there were few methylations in any of the miRNAs in either the *Hp*−/AG or the *Hp*−/GC group, and there was no significant difference in the incidence of miRNA methylation between the two groups. However, the *miR-129-2* methylation rate in the angulus was significantly higher in the *Hp*−/GC group than in the *Hp*−/AG group (*p* = 0.01) (Fig. 1B).

Molecular events in IM: The incidence of all miRNA genes in IM was demonstrably higher than in non-IM regardless of the presence or absence of *H*. *pylori* infection in both the AG and GC groups (Table 3), a finding that was consistent with our previous report^33,34^. Therefore, there were no significant differences in the methylation rates for each miRNA gene among the four groups, i.e., the *Hp*+/AG, *Hp*−/AG, *Hp*+/GC, and *Hp*−/GC groups. Furthermore, no significant differences in the incidence of miRNA gene methylation were seen between the AG and GC groups in each part of the stomach in both *H*. *pylori*-infected and -eradicated patients, unlike in non-IM (Supplementary Table S1).

**Table 3:** Changes of molecular alterations in IM by *H*. *pylori* eradication (Cohort 1).

	*Hp*+/AG group		*Hp*−/AG group		p–value	*Hp*+/GC group		*Hp*−/GC group		p–value	*Hp*+/AG group vs *Hp*+/GC group	*Hp*−/AG group vs *Hp*−/GC group
	(%)	(n)	(%)	(n)		(%)	(n)	(%)	(n)		p–value	p–value
*miR–34c*	61.9	(13/21)	54.5	(12/22)	0.63	56.0	(14/25)	58.8	(20/34)	0.83	0.77	0.79
*miR–124a–3*	100	(21/21)	86.4	(19/22)	0.23	83.3	(25/30)	88.6	(31/35)	0.72	0.07	1
*miR–129–2*	100	(18/18)	100	(22/22)	1	100	(33/33)	100	(32/32)	1	1	1
*miR–137*	95.0	(19/20)	100	(21/21)	0.49	100	(34/34)	100	(34/34)	1	0.37	1

IM, intestinal metaplasia; *Hp*, *H*. *pylori*; AG, atrophic gastritis; GC, gastric cancer; OR, odds ratio; CI, confidence interval.

#### Cohort 2

**Figure 2:**
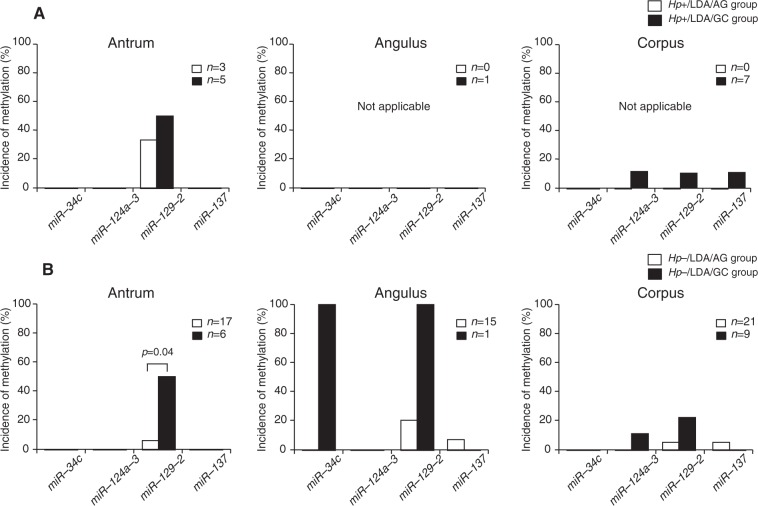
Incidences of miRNA methylation in non-IM in the three parts of the stomach in patients regularly taking LDA/NSAIDs. (**A**) In *H*. *pylori*-infected patients, there were no significant differences in the incidences of miRNA methylation in each portion of the stomach. (**B**) In *H*. *pylori*-eradicated patients, the *miR-129-2* methylation rate in the antrum was significantly higher in the *Hp*−/GC group than it was in the *Hp*−/AG group (*p* = 0.04). LDA, low-dose aspirin; NSAID, nonsteroidal anti-inflammatory drug.

Molecular alterations in non-IM: The incidence of miRNA methylation was not significantly different between the *Hp*+/LDA/AG and *Hp*−/LDA/AG groups or between the *Hp*+/LDA/GC and *Hp*−/LDA/GC groups (Table 4), a result that was different from the findings in Cohort 1. However, only *miR-129-2* methylation was more frequently observed in the *Hp*−/LDA/GC group than in the *Hp*−/LDA/AG group (*p* = 0.02) among the *H*. *pylori*-eradicated patients, although there were no significant differences in the incidences of the methylation of other miRNAs between the AG and GC groups regardless of the presence or absence of *H*. *pylori* infection. When looking at the methylation rate in each portion of the stomach, the *miR-129-2* methylation rate was found to be significantly higher in the *Hp*−/LDA/GC group than in the *Hp*−/LDA/AG (*p* = 0.04) group only in the antrum (Fig. 2B).

**Table 4:** Comparison of molecular alterations between patients taking LDA/NSAIDs with and without GC (Cohort 2).

Non-IM	*Hp*+/LDA/AG group		*Hp*−/LDA/AG group		*p*–value	*Hp*+/LDA/GC group		*Hp*−/LDA/GC group		*p*–value	*Hp*+/LDA/AG group vs *Hp*+/LDA/GC group	*Hp*−/LDA/AG group vs *Hp*−/LDA/GC group
	(%)	(*n*)	(%)	(*n*)		(%)	(*n*)	(%)	(*n*)		*p*–value	*p*–value
*miR–34c*	0	(0/3)	0	(0/53)	1	0	(0/13)	6.3	(1/16)	1	1	0.23
*miR–124a–3*	0	(0/3)	0	(0/53)	1	8.3	(1/12)	6.3	(1/16)	1	1	0.23
*miR–129–2*	33.3	(1/3)	9.4	(5/53)	0.29	25.0	(3/12)	37.5	(6/16)	0.69	1	0.02
*miR–137*	0	(0/3)	3.8	(2/53)	1	7.7	(1/13)	0	(0/16)	0.45	1	1
**IM**	**(%)**	**(** ***n*** **)**	**(%)**	**(** ***n*** **)**	***p*** **–value**	**(%)**	**(** ***n*** **)**	**(%)**	**(** ***n*** **)**	***p*** **–value**	***p*** **–value**	***p*** **–value**
*miR–34c*	40.0	(2/5)	60.0	(6/10)	0.61	75.0	(9/12)	58.3	(7/12)	0.67	0.28	1
*miR–124a–3*	80.0	(4/5)	100	(9/9)	0.36	94.4	(17/18)	93.3	(14/15)	1	0.40	1
*miR–129–2*	100	(4/4)	100	(7/7)	1	100	(11/11)	100	(10/10)	1	1	1
*miR–137*	100	(4/4)	100	(7/7)	1	100	(12/12)	100	(12/12)	1	1	1

LDA, low–dose aspirin; NSAID, nonsteroidal anti-inflammatory drug; *Hp*, *H*. *pylori*; AG, atrophic gastritis; GC, gastric cancer; IM, intestinal metaplasia.

Molecular alterations in IM: The incidences of methylation of all of the miRNA genes in IM were higher compared to those in non-IM, and they were not significantly different between the *Hp*+/LDA/AG and *Hp*−/LDA/AG groups or between the *Hp*+/LDA/GC and *Hp*−/LDA/GC groups, findings that were similar to the results in non-IM (Table 4). Similarly, there were no significant differences in the incidences of methylation of all miRNAs between the AG and GC groups regardless of the presence or absence of *H*. *pylori* infection. In addition, no positive associations in the incidence of miRNA gene methylation were seen between the AG and GC groups in each part of the stomach in either *H*. *pylori*-infected or -eradicated patients, as in IM in Cohort 1 (Supplementary Table S1).

Effects of long-term aspirin use: In *H*. *pylori*-infected patients, only *miR-34c* methylation in non-IM was significantly lower in the *Hp*+/LDA/GC group compared to the *Hp*+/GC group (*P* = 0.047), which may be an effect of long-term aspirin use. In contrast, there were no significant differences in other molecular events between patients who were taking and patients who were not taking LDA/NSAIDs in both non-IM and IM, irrespective of *H*. *pylori* infection (Table 5).

**Table 5:** Statistical comparison of the incidence of molecular events between patients taking and not taking LDA/NSAIDs.

Non-IM	*H*. *pylori*-infected patients		*H*. *pylori*-eradicated patients	
	*Hp*+/AG vs *Hp*+/LDA/AG	*Hp*+/GC vs *Hp*+/LDA/GC	*Hp*−/AG vs *Hp*−/LDA/AG	*Hp*−/GC vs *Hp*−/LDA/GC
*miR–34c*	1	0.047	1	1
*miR–124a–3*	0.54	0.08	1	1
*miR–137*	1	1	0.58	1
*miR–129–2*	0.32	0.50	0.70	0.08
**IM**				
*miR–34c*	0.62	0.31	1	1
*miR–124a–3*	0.19	0.39	0.54	1
*miR–137*	1	1	1	1
*miR–129–2*	1	1	1	1

Values indicate *p*–values.

LDA, low–dose aspirin; NSAID, nonsteroidal anti-inflammatory drug; IM, intestinal metaplasia; *Hp*, *H*. *pylori*; AG, atrophic gastritis; GC, gastric cancer.

## Discussion

To the best of our knowledge, this is the first study to show the effects of *H*. *pylori* eradication and LDA/NSAIDs on the methylation of several miRNAs in patients with and without GC.

### Cohort 1

In patients not taking LDA/NDAIDs, *H*. *pylori* eradication was able to reverse the methylation of most miRNA genes only in non-IM, but not in IM, both in patients with and in those without GC (i.e., the *Hp*+/AG and *Hp*+/GC groups). In multivariate analysis, *H*. *pylori* eradication was associated with a significant reduction of *miR-124a-3* methylation in both groups, a finding that was consistent with our previous study^34^. In addition, *miR-34c* and *miR-129-2* methylation were associated with GC development in *H*. *pylori*-infected cases, and only *miR-129-2* methylation in non-IM was an independent risk marker of significant GC.

When evaluating the differences in the methylation of miRNA genes in each of the three parts of the stomach, we found that *miR-34c* methylation in non-IM in the antrum and corpus portions and *miR-129-2* methylation in non-IM in the corpus might be useful biomarkers of GC in *H*. *pylori*-infected patients. Meanwhile, *miR-129-2* methylation in non-IM in the angulus was associated with GC development in *H*. *pylori*-eradicated patients, thus indicating that the biomarkers for GC differ for each part of the stomach according to *H*. *pylori* status. Suzuki *et al*. reported that *miR-34b/c* methylation in the corpus is an independent predictor of metachronous GC risk^37^. However, they did not investigate the methylation of miRNA genes in non-IM and IM separately in *H*. *pylori*-infected and -eradicated patients.

### Cohort 2

In patients taking LDA/NSAIDs, the incidence of miRNA gene methylation was not different between *H*. *pylori*-infected and -eradicated patients in either non-IM or IM. Meanwhile, *miR-129-2* methylation in non-IM was significantly involved in GC development in *H*. *pylori*-eradicated patients. Therefore, this result indicates that patients with *miR-129-2* methylation in non-IM, especially in the antrum, may be at risk of GC. In the present study, LDA/NSAIDs decreased *miR-34c* methylation in non-IM in *H*. *pylori*-infected patients with GC (Table 5). This result may suggest that the chemopreventive effect of aspirin is limited to non-IM, and does not occur in IM, in *H*. *pylori*-infected patients. Some studies showed in stratified analyses that the chemopreventive effect of aspirin was higher in *H*. *pylori*-infected subjects^42–44^; this finding was in agreement with our results from the viewpoint of miRNA analysis. However, as *H*. *pylori* status was judged using only *H*. *pylori* IgG antibody in those previous studies^42–44^, no precise diagnosis of *H*. *pylori* infection was performed. In addition, it may be possible that the *H*. *pylori*-negative subjects in those studies included two subtypes that were completely different in terms of their GC risk: *H*. *pylori*-uninfected patients and patients who were suspected of naturally eradicated *H*. *pylori*. In our study, we showed that the long-term use of LDA/NSAIDs did not affect the changes in miRNA methylation in either *H*. *pylori*-infected or -eradicated patients, although the number of samples evaluated may have been small. Cheung *et al*. recently reported a clinical study in which the protective effect of aspirin appeared to be larger in *H*. *pylori*-eradicated subjects (hazard ratio = 0.30)^45^, a finding that was different from our results. Therefore, the mechanism of the chemopreventive effect of aspirin cannot be explained by miRNA methylation alone.

In the present study, the methylation of tumor-suppressor miRNAs was identified more frequently in IM and very infrequently in non-IM. It thus appears that the methylation of these genes is a molecular event that occurs specifically in IM, and also that IM might exhibit a more aggressive state than non-IM with regard to molecular alterations, as shown in our previous reports^33,34^. Intriguingly, only in *H*. *pylori*-eradicated patients, the number of IM samples obtained from the three parts of the stomach was significantly higher in patients with GC (the *Hp*−/GC and *Hp*−/LDA/GC groups) than in those without GC (the *Hp*−/AG and *Hp*−/LDA/AG groups). This result may support the clinical fact that GC is related to the extent of IM throughout the stomach^46^, especially after *H*. *pylori* treatment. Also, *H*. *pylori* eradication and the long-term use of LDA/NSAIDs reversed the methylation of miRNAs in non-IM, but not in IM. Taken together, these results are in agreement with the concept of a “point of no return”^47^, which holds that the benefits of *H*. *pylori* eradication and aspirin diminish after the IM stage is reached through the state in which molecular changes are irreversible. AG is caused by focal inflammation, resulting in a loss of glandular structures in the gastric mucosa, while IM involves the replacement of damaged gastric mucosa by intestinal epithelium, including goblet cells and absorptive cells^48^. In the inflammatory state, gastric tissue stem cells fail to differentiate normally^49^, resulting in their progression to IM^50^. In addition, the appearance of IM is considered to be associated with the aberrant expression of CDX1 and CDX2^51,52^. Based on previous reports^48–52^ and the present study, the pathogenesis of non-IM and that of IM are likely to be different from the viewpoint not only of histology but also of the differentiation of gastric stem cells and the accumulation of molecular events. It has recently been reported that AG and IM were reversed by *H*. *pylori* treatment over the long term^48^, and the reversibility of IM was associated with a decrease in CDX2 mRNA expression^52^. Thus, a long-term follow-up study may be necessary to confirm the molecular changes resulting from such interventions in non-IM and IM cases.

The present study had a methodological advantage: as our data from LCM samples delineated many GC-related miRNA genes, this procedure might provide more information from non-IM and IM regarding pathogenesis than would the use of whole-tissue material^53^. Since whole-biopsy tissues were used for DNA methylation analysis in the previous studies^29,30,36–41^, the differences in methylation between non-IM and IM were not evaluated. Therefore, those results may be affected by the total volume of IM glands contained in the biopsy samples^34^.

Our investigation had some limitations. First, this was a study from a single institution with a small number of *H*. *pylori*-infected patients who were taking LDA/NSAIDs; the number of patients was especially small considering that molecular alterations in three different parts of the stomach were compared. The second limitation is that this study may have been biased due to our use of the same samples collected in our previous two studies^33,34^. Third, the methylation-sensitive high-resolution melting (MS-HRM) used in our study may be applicable for semiquantitative but not quantitative assessment of the methylation levels in an unmethylated background. However, we confirmed that the definition of methylation (>10%) by MS-HRM was reasonable on the basis of the correlation coefficient of the calibration curve derived from the fluorescence value of the melting curve using the methylation standard control DNA^34^. Fourth, a relatively high number of samples could not be analyzed for molecular alterations due to the small amount of DNA that was extracted from the very small biopsy specimen. Therefore, further prospective studies with larger sample sizes are needed to clarify the association between miRNAs and gastric carcinogenesis in the background mucosa with and without GC.

In conclusion, in patients who were not taking LDA/NSAIDs (Cohort 1), (1) *H*. *pylori* eradication was associated with a significant reduction of *miR-124a-3* methylation only in non-IM in the background mucosa with and without GC, but not in IM, and (2) *miR-129-2* methylation in non-IM, especially in the corpus, may be a surrogate marker of GC in *H*. *pylori*-infected patients. On the other hand, in patients regularly taking LDA/NSAIDs (Cohort 2), LDA/NSAIDs did not affect the changes in miRNA methylation in either non-IM or IM, irrespective of *H*. *pylori* infection. However, *miR-129-2* methylation in non-IM, especially in the antrum, was an independent predictive marker of GC in *H*. *pylori*-infected patients. These results indicate that *H*. *pylori* eradication and LDA/NSAIDs use were less effective in improving the methylation in IM compared with non-IM; thus, these interventions are recommended to prevent GC development in individuals at an early stage prior to the development of IM.

## Patients, Materials and Methods

### Cohort 1

#### Patients

We previously conducted a cross-sectional study to define the differences in molecular alterations in non-IM and IM before and after *H*. *pylori* eradication (≥3 yr) in patients with and without GC^28,33,34^. By using the same samples, we herein performed a case-control study on miRNAs in four groups of the same patients based on previous studies:^28,33,34^ patients with histologically diagnosed AG (GC-free patients) who were positive for *H*. *pylori* (*Hp*+/AG group, n = 21); GC patients with *H*. *pylori* infection (*Hp*+/GC group, n = 26); patients with histologically diagnosed AG (i.e., GC-free patients) who had undergone *H*. *pylori* eradication >3 years before and had AG in the background mucosa (*Hp*−/AG group, n = 30); and patients who developed primary early GC despite the successful eradication of *H*. *pylori* at >3 years before (*Hp*−/GC group, n = 27). Histological diagnosis of GC was made in accordance with the GC criteria of the Japanese Gastric Cancer Association^54^. Patients with a history of esophagectomy or gastrectomy and who were taking LDA or other NSAIDs were excluded.

#### H. pylori status and DNA extraction

In our previous studies^28,33,34^, three biopsy specimens were taken during an endoscopy from three parts of the stomach: the greater curvature at the antrum, the greater curvature at the corpus, and the lesser curvature at the angulus (one from each site). Each biopsy specimen was cut into 4-µm-thick tissue sections and subjected to histological analysis using hematoxylin and eosin staining and Giemsa staining. *H*. *pylori* status was analyzed as reported previously^28,33,34^. A patient was regarded as *H*. *pylori-*positive if the result of at least one of the three aforementioned methods, i.e., the urea breath test (UBT), Giemsa staining, and the E-plate anti-*H pylori* IgG antibody test (Eiken Kagaku, Tokyo, Japan), was positive. *H*. *pylori* status following the eradication was determined by the UBT at least 6 weeks or more after the end of the anti-*H*. *pylori* treatment. From the paraffin-embedded biopsy specimens, two or three 7-µm-thick tissue sections were cut for DNA extraction. Goblet IM glands were isolated using the PALM MicroBeam LCM system (Microlaser Technologies, Munich, Germany), and DNA was extracted from goblet IM (incomplete type) and non-IM using the QIAamp DNA Micro Kit (Qiagen, Hilden, Germany) as previously reported^28,33,34^ (Supplementary Fig. S1). Finally, a total of 307 biopsy samples from 104 patients, including *Hp*+/AG, *Hp*+/GC, *Hp*−/AG, and *Hp*−/GC patients, were analyzed. However, five samples could not be analyzed for molecular alterations due to the small amount of DNA that was extracted from the very small biopsy specimens.

#### Sodium bisulfite modification of DNA of miRNA genes

As in previous reports^28,33,34^, purified DNA samples were chemically modified with sodium bisulfite using an EpiTect^®^ Fast Bisulfite Kit (Qiagen). The bisulfite-modified DNA was amplified using primer pairs that specifically amplify the methylated or unmethylated sequences of several miRNAs related to carcinogenesis, including *miR-34c*, *miR-124a-3*, *miR-129-2*, and *miR-137*.

#### MS-HRM analyses

MS-HRM analysis was performed as previously described^28,33,34^. Briefly, polymerase chain reaction (PCR) amplification and MS-HRM analysis were performed using a LightCycler^®^ 480 System II (Roche, Mannheim, Germany). The primer sequences of all genes for the methylated and unmethylated forms and PCR and MS-HRM conditions are summarized in Supplementary Tables S2 and S3. Percentages of methylation (0%, 10%, 50%, and 100%) were used to draw the standard curve (Supplementary Fig. S2). In this study, only samples with >10% methylation were considered to be methylated, as reported previously^33,34^.

### Cohort 2

#### Patients and samples

In this study, which was based on our previous studies^28,33^, we analyzed 22 patients who had developed primary GC despite taking LDA (100 mg/day) or NSAIDs for more than 3 years. These patients included 11 *H*. *pylori*-infected patients (*Hp*+/LDA/GC group) and 11 *H*. *pylori*-eradicated patients (*Hp*−/LDA/GC group). We also analyzed 24 patients with histologically diagnosed AG (GC-free patients) who regularly took LDA or NSAIDs for more than 3 years, who included *H*. *pylori*-infected cases (n = 3, *Hp*+/LDA/AG group) and *H*. *pylori*-eradicated cases (n = 21, *Hp*−/LDA/AG group). Biopsy specimens were taken from the same portions of the stomach in the same manner as described for Cohort 1. Patients with a history of esophagectomy or gastrectomy were excluded.

In Cohort 2, the methylation of miRNAs was analyzed. A total of 137 biopsy samples from the 46 patients making up the *Hp*+/LDA/AG, *Hp*+/LDA/GC, *Hp*−/LDA/AG, and *Hp*−/LDA/GC groups were analyzed. However, one sample could not be analyzed for molecular alterations due to the small amount of DNA that was extracted.

#### Consent and institutional review board approval

Written informed consent was obtained from all patients prior to these studies. The Ethics Committee of Hyogo College of Medicine approved these trials (Nos. Rin-Hi 136 and 300). These trials were registered with the UMIN Clinical Trials Registry (No. UMIN000021857). The study was performed in accordance with the Declaration of Helsinki.

#### Statistical analysis

Categorical variables were presented as numbers and percentages and compared between groups using the chi-square test or Fisher’s exact test when appropriate. Continuous variables were expressed by median and interquartile range and compared between groups using the Kruskal-Wallis test or Mann-Whitney *U-*test. Predictive factors for GC with a *p-*value of <0.05 in univariate analysis were included in the multiple logistic regression model and analyzed using the backward approach. ORs and 95% CIs were calculated for risk factors. Multivariate logistic regression analysis was performed to identify the molecular alterations associated with GC if a *p*-value of less than 0.1 in the univariate analysis was identified. A two-tailed *p*-value less than 0.05 was considered significant. Statistical analyses were performed with SPSS 22.0 (SPSS Inc., Chicago, IL) and StatView version 5.0 (SAS Institute Inc., Cary, NC).

## Supplementary information

Supplementary Figures and Tables
