# Supplementary material for: DNA methylation silencing of microRNA gene methylator in the precancerous background mucosa with and without gastric cancer: Analysis of the effects of H. pylori eradication and long-term aspirin use
Source: Sci Rep. 2019 Aug 29;9:12559. doi: 10.1038/s41598-019-49069-1 (PMC6715663; doi:10.1038/s41598-019-49069-1)
Supplement: Supplementary file 1 — Supplementary Figures and Tables [file 41598_2019_49069_MOESM1_ESM.docx]

**DNA methylation silencing of microRNA gene methylator in the precancerous background mucosa with and without gastric cancer: Analysis of the effects of *H. pylori* eradication and long-term aspirin use**

Jiro Watari ^1^, Chiyomi Ito ^1^, Tadakazu Shimoda ^2^, Toshihiko Tomita ^1^, Tadayuki Oshima ^1^, Hirokazu Fukui ^1^, Das KM ^3^, Hiroto Miwa ^1^

^1^ Division of Gastroenterology, Department of Internal Medicine, Hyogo College of Medicine, Nishinomiya, Japan; ^2^ Division of Pathology, Shizuoka Cancer Center, Shizuoka 411-8777, Japan; ^3^ Division of Gastroenterology and Hepatology, Departments of Medicine and Pathology, Robert Wood Johnson Medical School, Rutgers, Cancer Institute of New Jersey, New Brunswick, New Jersey 08903, United States

**Supplementary Figure S1.** (A) The glands of incomplete-type intestinal metaplasia (IM) were isolated by laser microdissection. (B) The same section after the removal of metaplastic glands.

**Supplementary Figure S2.** Representative results of MS-HRM analysis for methylation. Results are shown for the *miR-129-2* gene, with positive (fully methylated) and negative controls (fully unmethylated). The melting peaks were calculated from melting curves of MS-HRM. Each sample was directly compared with its control to identify the sample’s methylation status, and the differences in fluorescence between samples were normalized by the analysis algorithms. Methylated and partially methylated DNA (≥ 10%) were considered to be positive for methylation, and unmethylated DNA was treated as negative. The sample in IM in the GC group shows a moderate level of methylation (≥ 50%).

**Supplementary Table S1.** Incidences of miRNAs methylation in intestinal metaplasia in three different parts of the stomach

1) *H. pylori*–infected patients without taking LDA/NSAIDs

|  | Antrum | | | | |  | Angulus | | | | |  | Corpus | | | | |
| --- | --- | --- | --- | --- | --- | --- | --- | --- | --- | --- | --- | --- | --- | --- | --- | --- | --- |
|  | *Hp*+/AG | | *Hp*+/GC | |  |  | *Hp*+/AG | | *Hp*+/GC | |  |  | *Hp*+/AG | | *Hp*+/GC | |  |
|  | (%) | (*n*) | (%) | (*n*) | *p*–value |  | (%) | (*n*) | (%) | (*n*) | *p*–value |  | (%) | (*n*) | (%) | (*n*) | *p*–value |
| *miR–34c* | 83.3 | 5/6 | 50.0 | 5/10 | 0.31 |  | 33.3 | 4/12 | 75.0 | 9/12 | 0.10 |  | 0 | 0/3 | 33.3 | 1/3 | 1 |
| *miR–124a–3* | 100 | 6/6 | 90.0 | 9/10 | 1 |  | 100 | 12/12 | 81.3 | 13/16 | 0.24 |  | 100 | 3/3 | 50.0 | 2/4 | 0.43 |
| *miR–129–2* | 100 | 6/6 | 100 | 12/12 | 1 |  | 100 | 11/11 | 94.4 | 17/18 | 1 |  | 100 | 1/1 | 100 | 3/3 | 1 |
| *miR–137* | 100 | 6/6 | 100 | 12/12 | 1 |  | 91.7 | 11/12 | 94.4 | 17/18 | 1 |  | 100 | 2/2 | 100 | 4/4 | 1 |

2) *H. pylori*–eradicated patients without taking LDA/NSAIDs

|  | Antrum | | | | |  | Angulus | | | | |  | Corpus | | | | |
| --- | --- | --- | --- | --- | --- | --- | --- | --- | --- | --- | --- | --- | --- | --- | --- | --- | --- |
|  | *Hp*–/AG | | *Hp*–/GC | |  |  | *Hp*–/AG | | *Hp*–/GC | |  |  | *Hp*–/AG | | *Hp*–/GC | |  |
|  | (%) | (*n*) | (%) | (*n*) | *p*–value |  | (%) | (*n*) | (%) | (*n*) | *p*–value |  | (%) | (*n*) | (%) | (*n*) | *p*–value |
| *miR–34c* | 60.0 | 3/5 | 50.0 | 7/14 | 1 |  | 53.3 | 8/15 | 62.5 | 10/16 | 0.72 |  | 50.0 | 1/2 | 75.0 | 3/4 | 1 |
| *miR–124a–3* | 100 | 5/5 | 85.7 | 12/14 | 1 |  | 86.7 | 13/15 | 88.2 | 15/17 | 1 |  | 50.0 | 1/2 | 100 | 4/4 | 0.33 |
| *miR–129–2* | 100 | 5/5 | 100 | 11/11 | 1 |  | 100 | 15/15 | 100 | 17/17 | 1 |  | 100 | 2/2 | 100 | 4/4 | 1 |
| *miR–137* | 100 | 4/4 | 100 | 13/13 | 1 |  | 100 | 15/15 | 100 | 17/17 | 1 |  | 100 | 2/2 | 100 | 4/4 | 1 |

3) *H. pylori*–infected patients with regularly taking LDA/NSAIDs

|  | Antrum | | | | |  | Angulus | | | | |  | Corpus | | | | |
| --- | --- | --- | --- | --- | --- | --- | --- | --- | --- | --- | --- | --- | --- | --- | --- | --- | --- |
|  | *Hp*+/LDA/AG | | *Hp*+/LDA/GC | |  |  | *Hp*+/LDA/AG | | *Hp*+/LDA/GC | |  |  | *Hp*+/LDA/AG | | *Hp*+/LDA/GC | |  |
|  | (%) | (*n*) | (%) | (*n*) | *p*–value |  | (%) | (*n*) | (%) | (*n*) | *p*–value |  | (%) | (*n*) | (%) | (*n*) | *p*–value |
| *miR–34c* | – | – | 100 | 1/1 | – |  | 33.3 | 1/3 | 85.7 | 6/7 | 0.18 |  | 0 | 0/2 | 66.7 | 2/3 | 0.40 |
| *miR–124a–3* | – | – | 100 | 5/5 | – |  | 100 | 3/3 | 88.9 | 8/9 | 1 |  | 50.0 | 1/2 | 100 | 4/4 | 0.33 |
| *miR–129–2* | – | – | 100 | 4/4 | – |  | 100 | 3/3 | 100 | 5/5 | 1 |  | 100 | 1/1 | 100 | 2/2 | 1 |
| *miR–137* | – | – | 100 | 4/4 | – |  | 100 | 3/3 | 100 | 5/5 | 1 |  | 100 | 1/1 | 100 | 3/3 | 1 |

|  | Antrum | | | | |  | Angulus | | | | |  | Corpus | | | | |
| --- | --- | --- | --- | --- | --- | --- | --- | --- | --- | --- | --- | --- | --- | --- | --- | --- | --- |
|  | *Hp*–/LDA/AG | | *Hp*–/LDA/GC | |  |  | *Hp*–/LDA/AG | | *Hp*­/LDA/GC | |  |  | *Hp*–/LDA/AG | | *Hp*–/LDA/GC | |  |
|  | (%) | (*n*) | (%) | (*n*) | *p*–value |  | (%) | (*n*) | (%) | (*n*) | *p*–value |  | (%) | (*n*) | (%) | (*n*) | *p*–value |
| *miR–34c* | 50.0 | 2/4 | 100 | 3/3 | 0.43 |  | 66.7 | 4/6 | 42.9 | 3/7 | 0.59 |  | – | – | 50.0 | 1/2 | – |
| *miR–124a–3* | 100 | 4/4 | 100 | 3/3 | 1 |  | 100 | 5/5 | 90.0 | 9/10 | 1 |  | – | – | 100 | 2/2 | – |
| *miR–129–2* | 100 | 3/3 | 100 | 2/2 | 1 |  | 100 | 4/4 | 100 | 6/6 | 1 |  | – | – | 100 | 2/2 | – |
| *miR–137* | 100 | 3/3 | 100 | 3/3 | 1 |  | 100 | 4/4 | 100 | 7/7 | 1 |  | – | – | 100 | 2/2 | – |

4) *H. pylori*–eradicated patients with regularly taking LDA/NSAIDs

LDA, low–dose aspirin; NSAID, nonsteroidal anti-inflammatory drug; *Hp*, *H. pylori*; AG, atrophic gastritis; GC, gastric cancer.

Supplementary Table S2. Primer sequences for the MS-HRM assays

| Gene | Primer sequences (5’ – 3’) |
| --- | --- |
| *miR34c* | F- GATTGTATTGTGGTGGTTATAATTATTAAT |
|  | R- CCTCCAAAAATTTTACTTTCCTAAC |
| *miR124a-3* | F- GGGAGAAGTGTGGGTTTTTT |
|  | R- CCTTAATTATATAAACATTAAATCAAAATC |
| *miR129-2* | F- GGAGATATTTTGGGTTGAAGG |
|  | R- AAATTATATACAACAAACCCAAACC |
| *miR137* | F- ATTTTTTAGTTTTGGTTATTAGAGG |
|  | R- ACCAAAATAACACTTTCTTATTCTTTTC |

| Pre-incubation | | |  |  | Amplification | | | | | |  |  | Cooling | | |
| --- | --- | --- | --- | --- | --- | --- | --- | --- | --- | --- | --- | --- | --- | --- | --- |
| Denaturation (°C) | Hold  (min) | Ramp rate  (°C/s) |  |  | Target  (°C) | Hold  (sec) | Ramp rate  (°C/s) | Sec target^*^  (°C) | Step size^*^  (°C) | Step delay^*^  (°C) | Cycles |  | Target  (°C) | Hold  (sec) | Ramp rate  (°C/s) |
| 95 | 10 | 4.8 |  | Denaturation | 95 | 15 | 4.8 |  |  |  | 45 ^c^  48 ^d^  52 ^a,b^ |  | 40 | 30 | 2.5 |
|  |  |  |  | Annealing | 59 ^b^  60 ^a^  65 ^c,d^ | 30 | 2.5 | 54 ^b^  55 ^a,d^  59 ^c^ | 0.5 | 1 |  |  |  |  |  |
|  |  |  |  | Extension | 72 | 10 ^c,d^  30 ^a,b^ | 4.8 |  |  |  |  |  |  |  |  |
|  |  |  |  |  |  |  |  |  |  |  |  |  |  |  |  |
|  |  |  |  |  | High-resolution melting | | | | | |  |  |  |  |  |
|  |  |  |  | Denaturation | 95 | 60 | 4.8 |  |  |  |  |  |  |  |  |
|  |  |  |  | Annealing | 40 | 60 | 2.5 |  |  |  |  |  |  |  |  |
|  |  |  |  | Melting interval | 65 | 1 | 4.8 |  |  |  |  |  |  |  |  |
|  |  |  |  | Continuous | 95 | - | 0.02 | Acquisition 25 (/°C) | | |  |  |  |  |  |

**Supplementary Table S3.** PCR and MS-HRM conditions in each microRNA gene

^*^ Touchdown method, ^a^ *miR34c*, ^b^ *miR124a-3*, ^c^ *miR129-2,* ^d^ *miR137*
